# Modeling Adoption, Security, and Privacy of COVID-19 Apps: Findings and Recommendations From an Empirical Study Using the Unified Theory of Acceptance and Use of Technology

**DOI:** 10.2196/35434

**Published:** 2022-09-14

**Authors:** Nuno Nunes, Greta Adamo, Miguel Ribeiro, Bruna R Gouveia, Elvio Rubio Gouveia, Pedro Teixeira, Valentina Nisi

**Affiliations:** 1 Instituto Superior Técnico University of Lisbon Lisbon Portugal; 2 Interactive Technologies Institute / Laboratory for Robotics and Engineering Systems Lisbon Portugal; 3 Escola Superior de Enfermagem de São José de Cluny Funchal Portugal; 4 Regional Directorate of Health Regional Secretariat for Health and Civil Protection Governo Regional da Madeira Funchal Portugal; 5 University of Madeira Funchal Portugal; 6 Decipad New York City, NY United States

**Keywords:** COVID-19, SARS-CoV-2, UTAUT, empirical study, structural equation modeling, confirmatory factor analysis, security, privacy, global health, technology solution, research model, technology acceptance, digital health, mobile health

## Abstract

**Background:**

The global health crisis caused by COVID-19 has drastically changed human society in a relatively short time. However, this crisis has offered insights into the different roles that such a worldwide virus plays in the lives of people and how those have been affected, as well as eventually proposing new solutions. From the beginning of the pandemic, technology solutions have featured prominently in virus control and in the frame of reference for international travel, especially contact tracing and passenger locator applications.

**Objective:**

The objective of this paper is to study specific areas of technology acceptance and adoption following a unified theory of acceptance and use of technology (UTAUT) research model.

**Methods:**

We presented a research model based on UTAUT constructs to study the determinants for adoption of COVID-19–related apps using a questionnaire. We tested the model via confirmatory factor analysis (CFA) and structural equation modeling (SEM) using travelers’ data from an insular tourist region.

**Results:**

Our model explained 90.3% of the intention to use (N=9555) and showed an increased understanding of the vital role of safety, security, privacy, and trust in the usage intention of safety apps. Results also showed how the impact of COVID-19 is not a strong predictor of adoption, while age, education level, and social capital are essential moderators of behavioral intention.

**Conclusions:**

In terms of scientific impact, the results described here provide important insights and contributions not only for researchers but also for policy and decision makers by explaining the reasons behind the adoption and usage of apps designed for COVID-19.

## Introduction

### Background

There have been many papers addressing COVID-19–related impacts; more than 23,000 papers have been published between January and May 2020, and hence, it has proved difficult to remain up to date with all the released studies [1]. However, it is evident to all of us that the global health crisis caused by COVID-19 [2] has drastically changed human society in a relatively short time.

The pandemic’s socioeconomic impacts [3] are unprecedented (eg, in the education sector, more than a billion students were affected due to schools’ closure [3,4]). This situation created stress, especially for low-income families [3]. The COVID-19 pandemic has severely challenged the health care sector, especially medical workers severely exposed to physical and psychological repercussions [5]. In addition, people of color and other minorities have experienced more severe COVID-19 impacts than others due to socioeconomic conditions, health care disparities, and a lack of privileges [6,7]. In summary, the ongoing pandemic has disclosed to the world and exacerbated problems and inequalities based on gender, age group, ethnicity, socioeconomic situation, and nationality [8]. Supporting communities to promote well-being, social cohesion, and safe behaviors, especially for vulnerable groups, are welcome suggestions. However, governments and health institutions play an essential role in supporting well-being and providing economic, social, and health services and fostering trust [9].

The pandemic has challenged our progress and growth-based society and its capitalistic nature, and tourism, as a growth-based phenomenon, has suffered from these challenges [10]. However, the COVID-19 situation that has been ongoing for more than 2.5 years has provided an impetus to imagine and shape futures [11] by addressing existing problems, exploring new solutions to local and global challenges, and understanding the role of COVID-19 in affecting and changing people’s lives. Although much effort was made to develop and deploy several COVID-19 contact-tracing mobile apps [12,13], these technologies have raised several ethical challenges (eg, privacy, security, surveillance) [14-16] and their adoption has not been as expected [17], as the privacy policies have negative impacts on users’ privacy worries and the elements influencing personal benefits are greater than the community interests and outcomes when adopting an app. In addition, although technologies for citizen engagement have been considered helpful to manage crises [18], there is still a lack in this research area concerning COVID-19.

The general aim of this paper is to examine users’ perceptions and attitudes toward a COVID-19–based app through a case study on a European island, which deployed a successful safety system to mitigate the impact of the pandemic, while preserving mobility after lockdown and isolation. More specifically, the research aims of this work are (1) to investigate the effects of the COVID-19 pandemic on technology adoption and especially safety, security, privacy, and trust; (2) to increase our understanding of differences in the determinants of safety in technology use; and (3) to improve the predictive accuracy and explanatory power of a parsimonious questionnaire based on a known unified theory of acceptance and use of technology (UTAUT) [19] model for broader application in human-computer interaction (HCI) research.

A vital component of this research’s successful execution was the evaluation’s contained and isolated nature (ie, small European island with an extensive tourism economy [20]), which enabled rapid mobilization of research in tandem with the deployment of COVID-19 security measures. By designing and performing this research, we got an opportunity to analyze the near future in which safety tech apps will be 1 of the best attempts to deal with this “new normal,” and we collected data from an international audience recovering from the pandemic’s first wave.

However, the urgency to study COVID-19 phenomena could increase errors in the research and then decrease both rigor and validity. To avoid making such mistakes, we designed and distributed a questionnaire based on the UTAUT model [19] and collected data from 9555 participants from different nationalities. We applied exploratory and confirmatory factor analysis (CFA) and structural equation modeling (SEM) to analyze the data. The results of this study contain several implications for HCI research and COVID-19 tech design. The empirical findings demonstrate the validity of parsimonious assessment in evaluating a UTAUT-based model to understand the adoption and usage of the deployed safety app. Safety concerns and willingness to follow precaution measures are strong predictors of the intention to use, which also affects security. Privacy is a central concern that needs to inform the design of safety apps. Our results are valid across the moderating roles of demographics, such as gender, age, and social capital.

The rest of this paper is organized as follows: We start by providing an overview of the current literature pertinent to this study. Next, we describe the research questions, hypotheses, and methods adopted for this study and the results. The work outcomes are analyzed and discussed, and the limitations of the research are presented, also considering the particular context of the research, the COVID-19 pandemic. At the end of the paper, we present the conclusion and future works.

### Literature Review

This section presents the background and literature review related to the main topics of this paper. The first subsection provides an overview on the transformations of the tourism sector and research caused by COVID-19; the second subsection deals with the technological measures and their ethical challenges involved with the COVID-19 pandemic; the third subsection touches on citizen engagement and social capital studies also in the context of COVID-19; and finally, the last subsection surveys the technology adoption scales and methodology that we used and extended in our study.

#### Tourism Transformations in Times of COVID-19

One of the sectors most scarred by the COVID-19 pandemic crisis is tourism [3,21]. According to a United Nations World Tourism Organization’s (UNWTO) report from May 2020 [22], the health crisis was associated with the 22% less international arrivals in tourist destinations during the first quarter of 2020 and threatened many tourism jobs. This led to substantial policy measures [23] to support Europe’s tourism, which is an essential source of income for many countries.

As described by Sigala [10], the COVID-19 pandemic has challenged the capitalistic society in which often tourism is embedded. Nevertheless, COVID-19 can also be seen as an occasion for “slowing down” [24] to criticize the current state of affairs [25] and explore transformations by reimagining and redesigning tourism [10] toward more sustainable alternatives [26], community-oriented initiatives, and “socialized” tourism [27,28]. Zenker and Kock [29] suggested some possible directions for the tourism research agenda involving COVID-19: (1) to address the complexity of the current pandemic and trace relationships among different impacted areas and involved variables; (2) to consider the possible drifts in the “destinations' images” based on the pandemic history of the destination itself; (3) to examine behavioral changes in the visitors (eg, changes in travel choices), (4) locals (eg, in-group and out-group dynamics between locals and visitors), and (5) the tourism sector (eg, increase collaborations among different sectors); and finally (6) to predict and assess the long-term and secondary consequences of COVID-19 in tourism, such as observing the change in priorities in the sector.

The redesigning in the tourism sector could also benefit from the use of COVID-19 technologies. An invitation for a change in the domain of e-tourism research has been made [30] to reinvent the field from an ontological and epistemological perspective. As mentioned by Gretzel et al [30], although technology solutions are powerful catalysts for transformations and have been already used in the tourism research and sector, e-tourism research should reflect on COVID-19, look at the future, and be reshaped following the principles of historicity, reflexivity, transparency, plurality, creativity, and, finally, social equity and diversity. All of them require different points of view and research fields to develop theories and interventions.

#### COVID-19, Technological Interventions, and Ethical Challenges

COVID-19 has also changed our relationship with technology [31]. Thanks to digital tools, we are able to monitor the evolution of the pandemic day by day (see, eg, [32-34]); to perform predictions based on models [35]; to participate in digital meetings, conferences, and classes; and to remain in contact with our loved ones [31].

Several tools have been developed and proposed to mitigate the risks associated with the COVID-19 and the spread of the disease and to perform diagnosis. Kumar et al [36] discussed different technologies (eg, artificial intelligence [AI]) used for several COVID-19 apps by dividing them into the following groups: (1) diagnosis using radiology images, (2) disease tracking, (3) health condition prediction, (4) computational biology, (5) protein structure prediction, (6) drug discovery, and (7) social awareness, web, and tech control. Whitelaw et al [37] provided a framework for describing the digital apps in response to COVID-19 (eg, planning, management, tracking, testing, and quarantine) by explaining their functionalities, the technology used, the countries that adopted these digital tools, and their respective advantages and disadvantages. Ting et al [38] reviewed the impact of several technologies (eg, AI, big data, internet of things [IoT]) in the service of health interventions for COVID-19 (eg, monitoring, surveillance, prevention, and diagnosis). Finally, Golinelli et al [39] provided a literature review that tackles the digital measures embraced by the health care system to manage COVID-19. One result of the study [39] outlines that diagnostic tools form the majority, followed by surveillance and prevention technologies.

Many surveys have been performed to classify and discuss contact-tracing apps [12,39,40]. Contact-tracing technology has been promptly identified as a powerful tool to control and mitigate the spread of the pandemic, and several frameworks exist, such as centralized, decentralized, and hybrid architectures, and various data management concerns populate the literature [12,39-41]. To deal with some of the differences and find common ground, in April 2020, the European eHealth Network developed a toolbox for the European states to follow, called *Mobile Applications to Support Contact Tracing in the EU Fight Against COVID-19 Common EU Toolbox for Member States* [42]. According to this document, the European Union (EU) apps should be compliant with some sociotechnical requirements: epidemiological (eg, inform the persons who have been at risk of contracting the virus), technical (eg, use of proximity technology), interoperability (eg, epidemiological alignment among member states), cybersecurity (eg, adoption of encryption), and safeguards (eg, voluntary-based app). The EU toolbox for contact tracing addresses further ethical challenges (eg, the importance of accessibility and inclusivity as fundamental rights to be preserved and protected in the development and deployment of such apps).

Due to the ethical issues involving COVID-19 digital tools, many authors have examined this dimension [14-16,43]. Tang [15] described and discussed concrete privacy-aware digital interventions for contact tracing, while Morley et al [43] proposed some ethical guidelines for the development and the deployment of tracking and tracing applications. The authors identified some general and universal principles (ie, necessity, proportionality, scientific soundness, and time-boundedness) and enabling conditions that influence the execution of the tools (eg, voluntariness, consent, anonymity, right to be forgotten, accessibility). In addition, Dubov and Shoptawb [14] considered the ethical challenges of contact-tracing technology; for example, the number of tests necessary for a practical contact-tracing app; deciding how to collect data; and issues on privacy, voluntariness and consent, transparency, and inclusion. The research reported by Gasser et al [16] was more general since they discussed the ethical and legal challenges of COVID-19 digital health tools (eg, symptom checkers, quarantine compliance), not just tracking and contact-tracing apps. Examples of these challenges are the validity and necessity of the research, privacy requirements, the autonomy of the users, possible discrimination risks, and the risk of repurposing retrieved data for other aims.

The users’ perception and acceptance of COVID-19 contact-tracing approaches were investigated by Lu et al [44] and Utz et al [45]. The former focused on participants’ perception of contact-tracing strategies (ie, digital apps and human contact tracing), with results that include aspects, for instance, of privacy, security, and accessibility and suggest both hybrid approaches for contact tracing that combines technological and human supports to strengthen values such as trust and transparency. The latter provided a study on users’ acceptance of COVID-19–tracing apps across countries (ie, Germany, the United States, and China), reporting that different places show different acceptances, preferences, and requirements. All these issues are closely connected with several ethical principles, such as autonomy, privacy, solidarity, and justice, that are fundamental to recommendations for COVID-19 digital tools.

#### Technologies, Citizen Engagement, and Social Capital

Despite the plethora of digital tools proposed for COVID-19 (see, eg, [12]), many are still debated due to ethical challenges and criticalities regarding user perceptions and preferences [46]. The development and importance of apps based on *citizen engagement* and *participation* in times of COVID-19 have been proposed as an alternative [47,48] since engagement and communication are critical factors for managing a crisis, as also identified by Chen et al [18] during this latest pandemic emergency, in which they studied the effect of the national health authority’s social media accounts on citizen engagement.

Public and citizen engagement is based on communication and building relationships between authorities and citizens, for instance, through dialogue and participation [18,49]. Today, digital platforms and near-universal access to mobile technologies have the power to support citizen engagement with governance and municipalities (see, eg, [50]), regarding pretty much any issue that relates to the citizens’ lives. Digital technologies can truncate citizen feedback loops with the government and enhance the implementation of public policy and improve citizen-municipality relationships [51]. For this reason, many self-service apps are being deployed, as many important cities have promoted mobile technologies [52]. Yet, self-service apps can be expensive and hard to deploy for smaller communities dealing with public funding [51]. Recently, the health care industry has focused on wearables devices [53,54], in which technology is seen as an enabler for self-prevention programs. However, the adoption, trust, and sustained use of these systems is challenging and involves critical and complex design considerations [53].

Digital technology for citizen engagement can also facilitate the development of *social capital* [55]. “Social capital” is a term that is commonly used but often poorly defined and conceptualized [56], yet it can be generally defined as the values of social relationships and networks that a person *has* in terms of membership [56,57]. As described by Mandarano et al [55], relationships, trust, and norms are the 3 elements that constitute social capital and can be increased with participation, collective actions, and decisions. Social capital and health are also connected, such as in the mortality rate and heart disease, especially when associated with one’s level of income [58,59]. Focusing specifically on COVID-19, the study of Borgonovi and Andrieu al [59] showed that communities with high social capital could be more prepared for COVID-19 also in terms of change in behaviors and isolation to protect other members. Another study [60] confirmed that social distancing measures alone are inadequate to mitigate COVID-19 spreading; instead, increasing a sense of community and consequently social capital is more effective in preventing the effects of the pandemic.

#### Technology Adoption, Its Privacy-Based Extensions and Applications

Models are widely used to study people’s intentions to adopt technology. The Technology Acceptance Model (TAM) [61] and UTAUT [19] were designed and tested to measure people’s tendency toward technology. TAM is derived from another popular theoretical framework called the theory of reasoned action (TRA) [62] that itself explains human behavior. TAM applies the TRA to explains users’ behavior and acceptance in reference to computer systems on the basis of the users’ attitudes/intentions, perceived usefulness and ease of use, and other variables. Although TAM is a useful theory, it has some flaws. Indeed, it does not include some important factors, such as the social and organizational contexts in which the technology is encountered [63]. To solve some of these issues [64,65], UTAUT was proposed by bringing together several user acceptance models, including TAM and the TRA. According to UTAUT [19], the following indicators are connected with the use of information technology: (1) performance expectancy (usefulness), (2) effort expectancy (ease of use), (3) social influence, and (4) facilitating conditions, which influence behavioral intention and use behavior, constituting the main predictors of behavioral intention.

As reviewed by Venkatesh et al [66], there are several applications, integrations, and extensions of the UTAUT paradigm. For instance, Khalilzadeh et al [67] and Shin [68] investigated security elements in the field of ecommerce and mobile payments by adopting UTAUT and extending it with security constructs, such as perceived security, perceived risk, and trust. Based on some of the definitions given by Khalilzadeh et al [67], Shin [68], and Mandrik and Bao [69] and adapting them for information systems, (1) perceived security is the user’s belief that an information system will be secure [67,68], (2) trust is defined as the user’s belief that the information system provider will satisfy the user’s needs and expectations [67,68], and (3) perceived risk is related to the sense of doubt or anxiety related to the (possible negative) final result of an action, behavior, or situation [67,69] associated with an information system.

The literature shows that users perceive as risky several of the so-called new products, so perceived risk has been often included in UTAUT [65,67,70]. For instance, Thakur and Srivastava [71] measured perceived risk, and their results confirmed their hypothesis stating that risk negatively influences the adoption intention of users. Focusing on trust, it has been shown that this aspect has an effect on performance and effort expectancy [72]. Trust also involves the users’ expectation concerning the compliance promise of the service provider; this aspect of trust is particularly important, especially in some domains in which the users are more vulnerable and then exposed to risks (eg, electronic financial transactions and medical care) [73,74]. As shown by Wilkowska and Ziefle [75], in the eHealth domain, privacy and security are central topics that influence the use and acceptance of technology. In a study conducted by Schnall et al [76], in the context of mobile health technologies, similar findings revealed that privacy (eg, access to information), security, and trust concerns do exist among users of such apps.

The UTAUT model, including its extensions, was also used in 2020 in the context of COVID-19 technologies. For instance, Békés and Aafjes-van Doorn [77] examined psychotherapists’ attitudes regarding web-based psychotherapy, also considering the new exigencies of the pandemic. Tiwari [78] focused their study on the adoption of university online classes. Finally, the research carried out by Chayomchai et al [79] centered on the use of technology by Thai people during the quarantine. We built on these efforts to extend the UTAUT scale to measure users’ attitudes in COVID-19 times.

### Research Questions and Hypotheses

This work was motivated by a unique set of circumstances to deploy safety measures at scale in a European island with a significant tourism industry in order to better understand the factors affecting the adoption and use of dedicated COVID-19 apps. We were particularly interested in investigating the role of safety, security, privacy, and trust in the context of the adoption of a voluntary COVID-19 app that supports air and sea access to an insular region. We also wanted to understand the effect of moderator variables (gender, age, education, and social capital) in the adoption of COVID-19 safety systems.

The *Madeira Safe to Discover* app was part of the COVID-19 safety mechanism designed by the local Health Authorities of Madeira Islands in order to achieve 2 main goals: to support travelers coming into the region by guiding them through the health requirements and to empower the health authorities with an information system that facilitates the monitoring and managing of the potential COVID -19 effects on the region. After the lockdown, the region opened borders, implementing a mandatory COVID-19 polymerase chain reaction (PCR) screening test. Travelers coming to the islands needed to present a valid COVID-19 test 72 hours before entry or be subject to testing upon entry. Registration of personal and travel details on the regional health system was mandatory, either manually through a form or by using the Madeira Safe to Discover system. Note that the use of the Madeira Safe to Discover app was neither a necessary requisite nor easier compared to the alternative (ie, the physical document); in fact, the travelers could choose either solution. After entering the region, travelers would undergo a voluntary 14-day vigilance period to submit an electronic daily health inquiry. The health authorities deployed a web-based Madeira Safe to Discover app to stimulate compliance with the safety procedures, since screening and monitoring procedures were constitutionally optional.

During their vigilance period, travelers received reminders for submitting their health inquiries via the Short Message Service (SMS). Those using the Madeira Safe to Discover app could receive their test results and submit their daily health inquiry electronically. In addition, they could decide to share their location while using the app voluntarily, but the system could not implement any automated contact-tracing mechanism. In summary, the Madeira Safe to Discover app is an optional digital tool that would improve COVID-19 safety measures for health authorities, while providing some practical benefits for travelers at their data expense.

The researchers involved in this study were asked to assist with the system’s design and advise on data protection and privacy issues, while producing an independent adoption and usage report. This set the stage to investigate at scale the effects of safety, privacy, and trust in the adoption of mobile apps and safety-monitoring systems.

More specifically, the research purposes of this work were (1) to investigate the effects of the COVID-19 pandemic on technology adoption, especially safety, security, privacy, and trust; (2) to increase our understanding of differences in the determinants of safety in technology use; and (3) to increase the analytical potential and predictive precision of a parsimonious questionnaire based on a known UTAUT model for broader application in HCI research.

**Figure 1:**
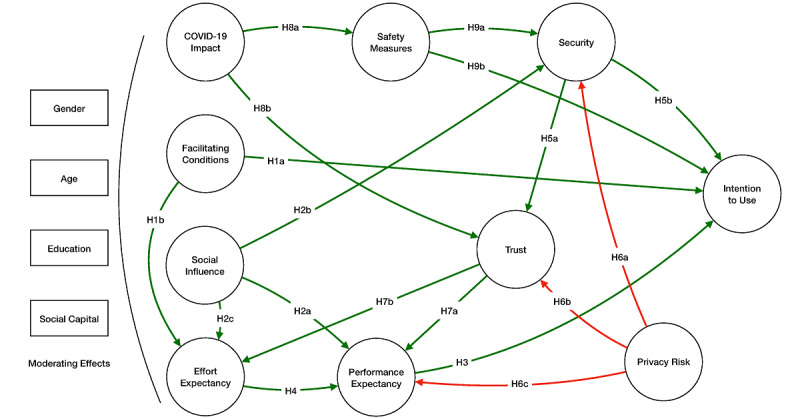
Proposed research model. H: hypothesis.

This study proposes a questionary adapted from a UTAUT model that incorporates variables such as safety, trust, perceived security, perceived usefulness (performance expectancy), and ease of use (effort expectancy). Figure 1 presents the Madeira Safe to Discover acceptance/use model proposed for this study.

For testing the hypothesis, the questionnaire comprised 27 questions (items) for responses on a Likert-type scale: 1 for strongly disagree, 2 for disagree', 3 for undecided, 4 for agree, and 5 for strongly agree. Concerning the questionnaire’s validity, the questions (items) were both adapted from the existent literature and reformulated considering the COVID-19 Madeira Safe to Discover app, which can generalized for safety-monitoring systems.

#### Items Based on UTAUT Constructs

For the purpose of this research, several hypotheses were developed on the basis of the original UTAUT constructs; we will lay them out in detail here.

*Facilitating conditions* are directly and positively related to user behavior but have no effect on behavioral intentions [19]. Our study followed the work of Khalilzadeh et al [67] in using behavioral intention as a surrogate for user behavior, although in the original UTAUT model, they are separate constructs. Therefore, we hypothesized that:

- Hypothesis 1a (H1a): The facilitating conditions (eg, owning a smartphone) for using the COVID-19 Madeira Safe to Discover app positively influences users’ intentions to use it.
- H1b: The facilitating conditions (eg, knowledge to use the app) for using the COVID-19 Madeira Safe to Discover app positively impacts effort expectancy.

*Social influence* directly and positively impacts behavioral intention. This means that people often discuss new technology with their friends, family members, and other people who are influential for them. These kinds of discussions could potentially produce changes in the opinions of the people concerning the new technologies. Following the argument founded by Khalilzadeh et al [67], focusing on the relationship between perceived security in the financial sector, we consider that perceived security is also central in health aspects; therefore, we assumed that perceived security should be relevant for the model. We thus hypothesized that:

- H2a: The social influence (eg, recommendation from significant others) for using the COVID-19 Madeira Safe to Discover app positively predicts effort expectancy.
- H2b: The social influence (eg, recommendation from significant others) for using the COVID-19 Madeira Safe to Discover app directly and positively influences perceived security.
- H2c: The social influence (eg, recommendation from health authorities) for using the COVID-19 Madeira Safe to Discover app directly and positively influences performance expectancy.

*Performance expectancy* is the most influential predictor of behavior intention [19]. As reported by Khalilzadeh et al [67], Yang [80] identified 2 kinds of performance expectancy, utilitarian performance expectancy and hedonic performance expectancy. Yet, according to Rodríguez and Trujillo [81], there is only a small effect of hedonic motivation. One of the characteristics of the Madeira Safe to Discover app is the ability to let travelers enter their own data and avoid queues and paper forms during an already stressful airport transit in pandemics’ context. For these reasons, since the app offers utilitarian benefits that could influence adoption, we hypothesized that:

- H3: Performance expectancy (ie, usefulness) positively affects behavioral intention to use the COVID-19 Madeira Safe to Discover app.

*Effort expectancy* is 1 of the most influential predictors of the intention to use mobile apps [82-84]. Others have also found effort expectancy to significantly impact behavioral intention [85,86]. Although in the original UTAUT model, effort expectancy affects the intention to use, the studies on which this research is based (eg [67,80]) in a departure from the original model posit that effort expectancy predicts performance expectancy.

As the Madeira Safe to Discover app provides a new way to secure travel, we expect that the perceived ease to use such an app will influence the behavioral intention of the users. Following the previous analyses and UTAUT’s hypotheses, we formulated that:

- H4: Effort expectancy (ie, ease of use) positively affects performance expectancy (ie, usefulness) to use the COVID-19 Madeira Safe to Discover app.

#### Items Based on UTAUT Extensions for Security and Privacy

Security, trust, and risk have become critical additional constructs in studies on technology adoption [65,67], especially in the case of sharing medical information.

*Perceived security* is supposed to directly affect behavioral intention [67]. Because the COVID-19 Madeira Safe to Discover app involves sensitive health information, we anticipated that perceived security would be influential in our model [67]. As stated by Khalilzadeh et al [67], perceived security is also an aggregate construct that changes over time and according to public opinion and social influence. Therefore, we hypothesized that:

- H5a: The perceived security of the COVID-19 Madeira Safe to Discover app positively and directly predicts perceived trust.
- H5b: The perceived security of the COVID-19 Madeira Safe to Discover app positively and directly predicts the behavioral intention to use the COVID-19 Madeira Safe to Discover app.

*Privacy risk* is usually associated with perceived security; the more a user senses privacy risks, the less secure they are likely to feel, leading to a negative relationship between risk and security [67,87]. Based on the findings retrieved from the literature (see the Literature Review section), which state that perceived risk has a negative impact on perceived security, trust, and performance expectancy, the following hypothesis were formulated:

- H6a: The privacy risk of using the COVID-19 Madeira Safe to Discover app directly and negatively impacts perceived security.
- H6b: The privacy risk of using the COVID-19 Madeira Safe to Discover app directly and negatively impacts perceived trust.
- H6c: The privacy risk of using the COVID-19 Madeira Safe to Discover app directly and negatively impacts performance expectancy.

*Trust*, together with perceived security, usually affects positively behavioral intentions [67,68]. Yet, considering only trust, its effect on behavioral intention has been considered significant [68,88]. As digital technologies become ubiquitous, trust supersedes more traditional technology adoption factors. Akin to Chandra et al [88], this study included trust as a singular construct. Hence, following Khalilzadeh et al [67] and Yang [80], we hypothesized that trust positively affects the effort expectancy and we formulated that:

- H7a: Trust positively impacts the performance expectancy (ie, usefulness) to use the COVID-19 Madeira Safe to Discover app.
- H7b: Trust positively affects the effort expectancy (ie, ease of use) to use the COVID-19 Madeira Safe to Discover app.

#### Items Related to the COVID-19 Impact and Safety Measures

The COVID-19 pandemic had a significant social, economic, and personal behavioral impact on citizens worldwide. Most countries in Europe were on complete lockdown for several weeks and months, and many closed airports and borders to prevent the spread of the pandemic. After COVID-19 lockdown, measures were enforced in public spaces (eg, use of masks, temperature screening, hand hygiene) to mitigate the risk of contagion. As introduced in the Literature Review section, technology adoption models are inspired by the TRA; according to this, subjective norms and the attitude toward an action impact the behavioral intention to use, so these 2 influence how individuals perform an action [62]. Adapted from the TRA and TAM, the UTAUT definition of attitude toward a behavior is “an individual’s positive or negative feeling about performing the target behavior” [19], while subjective norm refers to a “person’s perception that most people who are important to *them* think they should or should not perform the behavior in question” [19]. Therefore, we developed the following hypotheses:

- H8a: The extent to which someone is impacted by COVID-19 positively affects the intention to follow safety measures.
- H8b: The willingness to follow COVID-19 safety measures positively affects the intention to use the COVID-19 Madeira Safe to Discover app.

Furthermore, it is noteworthy that the attitude of a person concerning a particular behavior is dependent upon their beliefs as well as evaluations, and different works have stressed the relationship between security, safety, and behavioral intentions [65,67]. Despite the importance of trust and privacy risk in influencing the behavioral intention to use digital technologies, the current literature has not invested in understanding the role of perceived risk (eg, [73]). As travelers are likely to perceive the COVID-19 safety measures as risky, we expect that trust will play a significant secondary role in behavioral intention than privacy risk. However, trust might be more important in minimizing the risk perception. Hence, given the wide applicability of UTAUT, we can anticipate that:

- H9a: The willingness to follow COVID-19 safety measures positively and directly influences perceived security.
- H9b: The extent to which someone is impacted by COVID-19 positively and directly predicts perceived trust.

## Methods

### Study Design

This study followed the recommendation for a 2-stage analytical procedure [89]. To test the measurement model’s validity and reliability, we applied CFA, followed by SEM, to perform multiple regression analysis. CFA and SEM allow simultaneous analysis of both observed and latent variables, while providing overall fit statistics [90,91]. CFA was conducted using R v 4.0.2 (R Foundation for Statistical Computing) using maximum likelihood estimation. Path analysis of the structural relationships were also conducted using R with SEM libraries (lavaan v. 0.6-7 [92] and semTools v. 0.5-3 [93]). Moderation analysis [94] was also undertaken in R.

### Participants and Procedures

The questionnaire was sent via email to 58,954 participants who were registered in the system and who gave prior permission to be contacted via email. The questionnaire was sent at the end of August 2020 to travelers who had already finalized their trips or had stayed after the 14-day monitoring period (July and August 2020). The email was sent in all the 5 different languages supported by the app and contained a general explanation of the study, the details of the privacy policy and data treatment, and a link to a Google Forms survey. The questionnaire was translated into 5 languages corresponding to the supported idioms of the app according to the following breakdown: 36,930 (62.6%) in Portuguese (PT), 10,178 (17.3%) in English (EN), 6575 (11.2%) in German (DE), 3735 (6.3%) in French (FR), and 1536 (2.6%) in Spanish (ES). In total, we collected data from 9555 participants; corresponding to the overall participation of 16.2%, the participation was higher in DE (18.6%) and PT (17.7%) and lower in FR (12.2%), EN (11.6%), and ES (11.4%).

In terms of the general demographics (N=9555, summary in Table 1), the sample comprised a slightly higher proportion of women (n=5019, 52.5%) than men (n=4493, 47.0%), with 43 (0.5%) classifying themselves differently. There were a majority of Portuguese respondents (n=5847, 61.2%), followed by the major traditional tourism markets of Madeira Islands (n=1310, 13.7%, German; n=532, 5.6%, United Kingdom; n=516, 5.4%, French; n=328, 3.4%, Spanish; n=125, 1.3%, Italian), a few other EU (n=603, 6.3%) and other non-EU (n=125, 1.3%) markets, and a minority of 169 (1.8%) from non-European nationalities. In terms of age groups, young (<18 years old, n=142, 1.5%) and older (>65 years old, n=484, 5.1%) people were a minority compared to segments of the adult population (18-25 years old, n=3122, 32.7%; 18-25 years old, n=3203, 33.5%; 36-49 years old, n=2581, 27.0%). Finally, the sample was characterized with high education levels, with 70.4% holding a higher degree, 2307 (24.1%) having secondary education, and only 277 (2.9%) with basic education. The questionnaire also gathered some data on the frequency of travel, which is harder to characterize because of the different possible combinations between tourists, locals, and visitors. Nevertheless, surprisingly, 3726 (39.0%) respondents said it was their first time in Madeira, almost half of the respondents came regularly (n=4711, 49.3%), and 1080 (11.3%) said they were local residents. Note that the sample does not reflect the official tourism statistics, which changed drastically with the COVID19 pandemic. Indeed, the annual official statistics for2019 report that 87% of visitors were foreign (13% nationals), and of these, the majority were German (24%) and UK (23%) nationals.

The study took place within the scope of the Science4Covid Research project funded by the Portuguese National Science Foundation in collaboration with regional and national health authorities.

**Table 1:** Characteristics of respondents (N=9555).

Demographic and group			Frequency, n (%)
**Gender**			
	Woman	5019 (52.5)	
	Man	4493 (47.0)	
	Other	43 (0.5)	
**Age (years)**			
	<18	142 (1.5)	
	18-35	3122 (32.7)	
	36-49	3203 (33.5)	
	50-65	2581 (27.0)	
	>65	484 (5.1)	
	N/A^a^	23 (0.2)	
**Nationality**			
	Portuguese	5847 (61.2)	
	German	1310 (13.7)	
	United Kingdom	532 (5.6)	
	France	516 (5.4)	
	Spain	328 (3.4)	
	Italian	125 (1.3)	
	Other EU^b^	603 (6.3)	
	Other non-EU	125 (1.3)	
	Other (non-European)	169 (1.8)	
**Education**			
	Basic	277 (2.9)	
	Secondary	2307 (24.1)	
	Graduation	3686 (38.6)	
	Postgraduation	3035 (31.8)	
	N/A	250 (2.6)	

^a^N/A: not applicable.

^b^EU: European Union.

### Ethical Considerations

Given that the study did not involve sensitive or health-related information, did not involve risks or benefits, and was completely voluntary, it was not necessary to obtain an ethics board review. Nevertheless, the study complied with the provisions of the General Data Protection Regulation—Regulation (EU) 2016/279 of the European Parliament and of the Council of April 27, 2016—and follows the recommendations of the Declaration of Helsinki for research.

### Participants’ Motivations and Sources of Influence for Travel

Two questions addressed the main motivations and sources of influence for travel. Among the motivations for the trip, 1940 (20.3%) participants reported the sun, 1891 (19.8%) rest, 1749 (18.3%) nature, and 1491 (15.6%) family, followed by 1414 (14.8%) for COVID-19. Culture, work, and wellness were ranked much lower in terms of preference (n=612, 6.4%; n=325, 3.4%; n=134, 1.4%, respectively). In terms of nationality breakdown, family ranked higher for Portuguese nationals, while COVID-19 was higher for German and Spanish nationals. In terms of travel frequency, COVID-19 was almost equally higher for local residents and first-time visitors, which suggests that some people choose to travel to a destination because of COVID-19. This was confirmed by analysis of the sources of influence where safety had 3019 (31.6%) responses ranked first, followed by personal (n=2933, 30.7%) and family (n=2169, 22.7%) responses and a much lower influence on media, tour/agencies, and social media (n=812, 8.5%; n=401, 4.2%; and n=201, 2.1%, respectively). In terms of age, motivations were not significantly different, although COVID-19 consistently rose from 1041 (10.9%) for lower-age groups (<18 years) to 1815 (19.0%) for higher-age groups (>65). The same trend was not observed for safety in the sources of influence.

### Measurement Model

Inspired by the methodology described by Khalilzadeh et al [67], we examined the SEM assumptions by visually inspecting the variables shown in the diagrams, which ultimately appeared to have a normal distribution. In addition, the residuals manifested a normal distribution and no relationship was identified between predictors and residuals [75]. Focusing on the model itself (provided in Multimedia Appendix 1), its fits were good, reaching goodness-of-fit indices (GFIs) higher than the recommended thresholds of 0.8 for the adjusted goodness-of-fit index (AGFI) and 0.9 for other indexes [75,95-97].

Specifically, the GFI was 0.959, the AGFI was 0.928, the comparative fit index (CFI) was 0.959, the normative fit index (NFI) was 0.958, and the Tucker-Lewis index (TLI) was 0.950. Similarly, there was no misfit evidence, with satisfactory levels of 0.053 for the root-mean-square error of approximation (RMSEA) and 0.063 for the standardized root-mean-square residual (SRMR), which compared favorably to the benchmarks reported by Wilkowska and Ziefle [75], Fornell and Larcker [95], Bagozzi and Yi [96], and Etezadi-Amoli and Farhoomand [97], suggesting that values of 0.06 or less reflect a close fit. The SRMR was also good, at 0.063, below the overall fit threshold (<0.06). Due to the big sample size (N=9555), the model X^2^ was significant. After verifying the measurement fits of the data against the known thresholds, we built the initial measurement model to refine the questions and check the validity and reliability of the measurement items. All the loadings were significant at an *α* level of .001, with most factor loadings higher than 0.7 and 2 factors (impact and safety) slightly below the threshold at 0.470, indicating good convergent validity [95].

Table 2 shows the results of CFA. All items loaded significantly to the underlying constructs (*P*<.001), pointing to adequate convergent validity and reliability in all cases. We examined the convergent validity of the model by measuring the average variance extracted (AVE) and the reliability of each measure and each construct (provided in Multimedia Appendices 2 and 3 [98]). We compared the shared variance among constructs with the AVE from the individual construct to check discriminant validity (provided in Multimedia Appendices 2 and 3). Discriminant validity was checked by confirming that the heterotrait-monotrait ratio of correlations (HTMT) was below the 0.85 threshold [99]. Finally, the model was checked for composite reliability as an indicator of a latent construct of the shared variance among the observed variables. The composite reliability was 0.97, which indicated high measurement reliability of our measurement model [100].

In this model, we analyzed the moderating effect of the model factors and their effect on variables. In this sense, we can expect that the model will show unexpected moderating relationships [67]. In summary, we concluded that the measurement model exhibits good reliability and good convergent and discriminant validity.

**Table 2:** The measurement model.

Construct and item		*α*	SE	*Z* value	*P* value
**Impact of COVID-19 (Impact), *α*=.74**					
	Impact_1	.75	N/A^a^	N/A	N/A
	Impact_2	.76	0.015	58.294	.001
	Impact_3	.55	0.016	46.660	.001
**Facilitating conditions (FacCon), *α*=.92**					
	FacCon_1	.90	N/A	N/A	N/A
	FacCon_2	.94	0.009	117.527	.001
**Privacy risk (Privacy), *α*=.91**					
	Privacy_1	.92	N/A	N/A	N/A
	Privacy_2	.90	0.014	69.501	.001
**Social influence (SocInf), *α*=.60**					
	SocInfl_1	.65	N/A	N/A	N/A
	SocInfl_2	.61	0.024	44.3131	.001
**COVID-19 safety measures (Safety), *α*=.73, R^2^=0.446**					
	Safety_1	.74	N/A	N/A	N/A
	Safety_2	.62	0.021	50.066	.001
	Safety_3	.72	0.021	55.529	.001
**Effort expectancy (EffExp), *α*=.92, R^2^=0.628**					
	EffExp_1	.90	N/A	N/A	N/A
	EffExp_2	.93	0.007	136.111	.001
**Performance expectancy (PerfExp), *α*=.85, R^2^=0.757**					
	PerfExp_1	.89	N/A	N/A	N/A
	PerfExp_2	.85	0.010	105.849	.001
**Security, *α*=.91, R^2^=0.521**					
	Security_1	.84	N/A	N/A	N/A
	Security_2	.84	0.010	108.731	.001
	Security_3	.92	0.008	126.270	.001
**Trust, *α*=.85, R^2^=0.510**					
	Trust_1	.79	N/A	N/A	N/A
	Trust_2	.94	0.014	79.631	.001
**Behavioral intention (IntUse), *α*=.70, R^2^=0.903**					
	IntUse_1	.65	N/A	N/A	N/A
	IntUse_2	.82	0.015	66.639	.001

^a^N/A: not applicable.

### Structural Model

**Figure 2:**
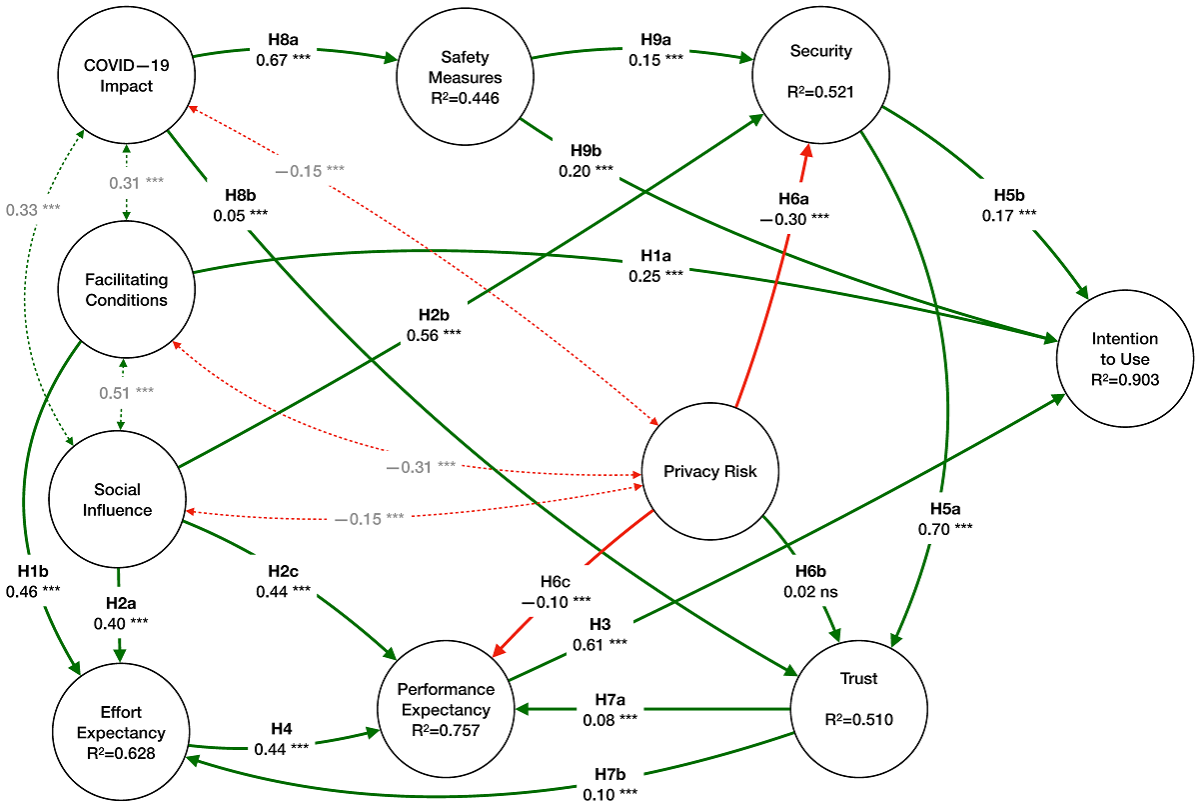
Results of the research model. H: hypothesis.

In the absence of measurement misfit, we applied SEM to perform multiple regression analysis of the data. This kind of technique is adopted to evaluate the fitting of the data upon the theoretical measurement model [68]. Here, we extended the proposed research model to include COVID-19–related constructs (COVID-19 impact and safety measures) and new interactions between these constructs and security, trust, and behavioral intention. The structural relationships were tested by estimating the causal paths defined by the hypotheses (see Figure 2 and Multimedia Appendix 4). All hypothesized causal paths, except H6b, were supported at *P*<.001. For all the constructs of the model, we calculated the squared multiple correlations (SMCs) represented as R^2^ in Table 2. This coefficient indicates the predictive accuracy and explanatory power of a model [75]. The SMC represents the share of the variance of the endogenous variable explained by the exogenous variables. The R^2^ value of the behavioral intention was the highest at 0.903, showing that the research model explains a large amount of the dependent variable variance. The lowest amount of R^2^ represented in the model was related to COVID-19 safety measures (R^2^=0.446), followed by trust (R^2^=0.510) and security (R^2^=0.521), due to the nature of the constructs (rooted in subject beliefs) and also their proximity to independent variables. The coefficient for performance and effort expectancy was also high at R^2^=0.757 and R^2^=0.628, respectively, consistent with previous results [67].

### Moderator Effects

To investigate demographic moderator effects, we followed the work of Shin [68], in which the split sample approach was adopted [101,102]. As described by Shin [68], the split sample approach is based on some moderators that are selected from the data and that cannot be changed. Some examples are a person’s nationality, gender, or age, which naturally form different moderator levels. We tested the moderator effects of gender (woman/man), age (divided into 2 groups, <36 and >36 years), education (basic/secondary education and higher education), and a proxy of social capital [56,57], which was calculated from a combination of nationality, residence, and regularity of travel. We classified local residents as high social capital, regular travelers or first-time national visitors as medium social capital, and first-time international visitors as low social capital.

We compared different groups to test the moderating effects of these variables after testing for measurement invariance using X^2^ difference tests and the fit indexes (provided in Multimedia Appendix 5). Invariance was also tested for factor structure, loadings, residuals, and means. The model supported good evidence of measurement invariance at *P*<.001 significance. The results of this analysis are presented in Table 3.

**Table 3:** Results of moderator effects.

Hypothesis	Gender		Age (years)			Education			Social capital		
	Woman	Man	<36	≥36	Basic/secondary		Higher education	Low		Medium	High
H1a	0.25^a^	0.23^a^	0.26^a^	0.25^a^	0.27^a^		0.25^a^	0.24^a^		0.24^a^	0.40^a,b^
H1b	0.43^a^	0.49^a^	0.45^a^	0.47^a^	0.51^a,c^		0.45^a^	0.49^a^		0.43^a^	0.52^a,c^
H2a	0.42^a^	0.37^a^	0.38^a^	0.41^a^	0.36^a^		0.40^a^	0.36^a^		0.41^a^	0.40^a^
H2b	0.60^a^	0.50^a,d^	0.51^a^	0.60^a^	0.62^a,c^		0.53^a^	0.52^a^		0.57^a^	0.57^a^
H2c	0.44^a^	0.48^a^	0.41^a^	0.47^a^	0.43^a^		0.45^a^	0.39^a,d^		0.43^a^	0.60^a,b^
H3	0.61^a^	0.62^a^	0.61^a^	0.61^a^	0.59^a^		0.61^a^	0.63^a^		0.63^a^	0.42^a,d^
H4	0.43^a^	0.43^a^	0.46^a^	0.41^a^	0.48^a^		0.42^a^	0.48^a^		0.45^a^	0.24 ^a,d^
H5a	0.68^a^	0.74^a^	0.71^a^	0.70^a^	0.71^a^		0.70^a^	0.67^a^		0.72^a^	0.71^a^
H5b	0.16^a^	0.19^a,c^	0.18^a^	0.16^a^	0.18^a^		0.17^a^	0.20^a,c^		0.13^a,d^	0.24^a,b^
H6a	–0.29^a^	–0.32^a^	–0.34^a,c^	–0.27^a,d^	–0.22^a,d^		–0.33^a^	–0.29^a^		–0.32^a^	–0.26^a,d^
H6b	0.02 (ns^e^)	0.01^a,d,e^	0^d,e^	0.03^b,e^	0.01^d,e^		0.02^e^	–0.02^d,e^		0.04^b,f^	0.02^b,e^
H6c	–0.11^a,c^	–0.07^a,d^	–0.09^a^	–0.11^a,c^	–0.08^a,d^		–0.10^a^	–0.10^a^		–0.10^a^	–0.08^a,g^
H7a	0.09^a,c^	0.05^d,g^	0.07^a^	0.08^a^	0.05^d,g^		0.09^a,c^	0.09^a,c^		0.07^a,d^	0.07^f^
H7b	0.10^a^	0.10^a^	0.12^a,c^	0.08^a,d^	0.09^a,d^		0.10^a^	0.10^a^		0.13^a,b^	0.01^d,e^
H8a	0.66^a^	0.67^a^	0.63^a^	0.70^a^	0.65^a^		0.68^a^	0.63^a^		0.65^a^	0.78^a,c^
H8b	0.06^a,b^	0.02^d,e^	0.03^d,e^	0.06^a,b^	0.05^f^		0.05^a^	0.04^d,f^		0.05^a,c^	0.04^d,e^
H9a	0.14^a^	0.15^a^	0.18^a,c^	0.11^a,d^	0.14^a^		0.15^a^	0.18^a,c^		0.12^a,d^	0.17^a,c^
H9b	0.15^a^	0.18^a,c^	0.14^a,d^	0.17^a^	0.16^a^		0.17^a^	0.17^a^		0.16^a^	0.08^d,g^

^a^Significant at *P*<.001.

^b^Highly significant increase in the *Z* value.

^c^Significant increase in the *Z* value.

^d^Significant decrease in the *Z* value.

^e^ns: not significant.

^f^Significant at *P*<.05.

^g^Significant at *P*<.01.

## Results

### Research Model Analysis

Results from the study demonstrated that our research model explains 90.3% of the intention to use the Madeira Safe to Discover app compared to previous research [67,103,104], which explained between 70% and 87% of the variance. Our model has stronger explanatory and predictive power, including new constructs related to the safety and personal impact of COVID-19, hence shaping a more complex network of interrelated causal relationships, which are not present in the original UTAUT and UTAUT2 models. This idea, borrowed from Khalilzadeh et al [67], which extends the UTAUT and UTAUT2 models with the inclusion of other influential constructs, increases the explicability of the model, while keeping parsimony. In line with some authors (eg, [100,105]), we reduced the number of items in some constructs, while preserving reliability, thus condensing the scale even further than previous research [67]. According to the recommendations of Worthington and Whittaker [67], we were able to retain factors with only 2 items, retaining validity, reliability, and correlation. The inclusion of the COVID-19 impact construct enabled us to understand whether there was a significant but weak impact on trust (H8b), especially when considering the moderation effects. Our results showed that for some groups (men, young people, and participants with some social capital on the premises), the COVID-19 impact on the user’s personal context is not significantly correlated to trust in the technology. The same weak link between the influence of trust on effort expectancy was illuminated for the group that had social capital on the premises, according to H7b. On the contrary, the role of COVID-19 safety measures in security (H9a) and behavioral intention (H9b) retained significance regardless of moderator variables. The effect of COVID-19 safety on security (H9a) decreased for old people and varied between groups that retained different social capital values at the arrival destination. Conversely, the role of COVID-19 safety in the intention to use (H9b) decreased for young people and people with higher social capital. In addition, results showed several significant relationships between the COVID-19 impact and several other constructs, which we did not hypothesize. These relationships showed stronger ties than our initial hypothesis on the COVID-19 impact and trust (Figure 2). Overall, the results demonstrate that the COVID-19 impact could be affected by facilitating conditions and social influence and it could influence privacy risk. Further research could highlight these effects. Interesting also was a negative influence of the COVID-19 impact on privacy, which we did not hypothesize.

Contrary to other empirical studies on mobile payments [67], our results showed a higher role of social influence in security and performance expectancy and a lower impact of privacy risk on performance expectancy and trust. Although for mobile payments, 67% of the variance in the security construct is explained by social influence (H2b) and risk perception (H6a), in our study, the variance explained was lower (52%) but social influence contributed more than privacy (H6a) and safety (H9a). Like Khalilzadeh et al [67], our results confirmed that users have severe concerns about their privacy and system performance. However, the impact of privacy was substantially reduced, which could be related to users’ compliance and acceptance of safety measures in general.

In terms of privacy and trust, our results differed significantly from previous studies [67,68]. The negative influence of risk on performance expectancy (H6c) was lower (from –0.25 to –0.10), and we could not confirm the hypothesis that privacy negatively impacts trust (H6b). We also observed negative correlations between perceived privacy and other constructs, which we did not hypothesize (COVID-19 impact, facilitating conditions, and social influence). Although some of these effects are reported in other studies on security and privacy [67-69], the COVID-19 impact on privacy should be further researched. In addition, the direct impact of social influence on security (H2b) was significant and robust and much higher than previous empirical research.

In addition to the COVID-19 impact, which is a new construct introduced here, security and privacy had a reduced impact on trust as well. Our results suggested that the impact of COVID-19 potentially affects privacy more than it does trust (1 of the unexpected results). Therefore, working on users’ privacy concerns is crucial for other similar COVID-19 systems since privacy influences perceived security and affects users’ trust toward these apps. Privacy also emerged as a more interrelated construct influencing performance expectancy and security but also showing significant relationships with the COVID-19 impact, facilitating conditions, and social influence. This clearly indicates that privacy needs to be addressed carefully while designing these apps and that its impact is not mitigated by the COVID-19 impact or the users’ willingness to follow safety measures.

Overall, the results indicated that performance expectancy (usefulness) is the biggest predictor of behavior intention to use (H3), which suggests that usability and ease of use are still crucial in designing COVID-19 systems. Effort expectancy was followed by facilitating conditions, COVID-19 safety measures, and, finally, security. Our results suggest that the willingness to follow COVID-19 safety measures (H9b) is a stronger predictor of usage behavior than security (H5b). This influence of H9a (Table 3) is stronger in young people and varies with different levels of social capital. These results suggest that special care should be taken to personalize apps for these groups when designing apps specific for COVID-19.

Finally, from all the moderator effects analyzed, clearly our indirect measure of social capital was the one showing more differences across the hypotheses. The predictors of the intention to use were significantly stronger for this group than any other group (Table 3), which suggests that designing an app targeting the local context will predict significantly higher adoption. Another relevant trend in the moderation of our hypothesis was the education level of the users, with lower education leading to fewer concerns about privacy (H2b) and security (H6a) but also less importance given to trust on performance (H7a) and effort (H7b) expectancy (also facilitating conditions).

## Discussion

### Principal Findings

The COVID-19 pandemic should be a stimulus to re-examine how we approach existing challenges (eg, social inequalities, sustainable tourism) and study some aspects of human behavior, such as our relationship with technology and its role during emergencies, for instance, in tourist destinations.

Against the backdrop of the COVID-19 pandemic, this paper provided the first detailed research on adopting mobile safety apps designed to mitigate the pandemic’s consequences. Although we expect that some of our findings will not be generalized beyond the context of the COVID-19 Madeira Safe to Discover app, others can provide early insight into the increasingly important role of safety, security, privacy, and trust in mobile app adoption and usage.

This research aimed at improving the predictive and explanatory power of technology use and adoption research models in the COVID-19 context. In addition, we investigated the variations in the determinants of COVID-19 systems’ acceptance in a reasonably diverse European demographic context.

The results from this work make apparent how privacy is a fundamental aspect when dealing with users’ perceptions of COVID-19–related systems. Indeed, privacy influences essential aspects, such as security and performance expectancy. Moreover, privacy concerns still stand, even when the impact of COVID-19 on the personal context of the user increases, showing the importance of privacy even in an emergency context. More generally, the impact of COVID-19 on people positively influences the adoption of safety measures (eg, use of masks, temperature screening, hand hygiene). Moreover, users who are more willing to follow COVID-19 safety measures are also more prone to using the COVID-19 Madeira Safe to Discover app. Several steps can be taken to further improve the usefulness of the app and ensure user trust and security, as was achieved with COVID-19 contact-tracing apps [106,107], although early receptibility proved to be low in some countries [108]. In Japan, the contact-tracing app COCOA [109] prioritized the protection of users’ privacy from a variety of parties, while enhancing the capacity to balance the current load of excessive pressure on health care systems, concluding in simulations that the participation rate in Japan needed to be close 90% to effectively control the spread of COVID-19. The COVID-19 Madeira Safe to Discover app proved to be well accepted by both citizens and visitors by not only recommending safe locations but also providing daily symptom inquiries and keeping that data available for the health authorities in following the design principle of electronic health records [110], being designed by considering the usability engineering [111] of the app and trustworthiness that was conveyed to the users, although this could be further improved by being even more transparent about how the data are processed, anonymized, and transmitted to the health authorities, showing the process in a data pipeline diagram.

Finally, this work’s fundamental contribution is an increased understanding of the essential role of privacy, security, and trust in the intention to use safety apps. Although security has a strong, direct and indirect effect on the model’s fundamental construct, it emerges to be as equally important as safety concerns. Furthermore, our research shows an increased role of social influence in security, of security in trust, and of trust in performance expectancy compared to previous research that inspired our model. Conversely, we observed a reduced negative impact of privacy on security and a rejection of the hypothesis of the positive role of privacy on trust compared to previous research. Together with a more complex influence of privacy on the overall model, these are significant results for future research implications.

### Limitations

Despite the contributions described previously, this research had some limitations, which also provide useful avenues for additional research discussed in the next section. Here, we reported on 1 of the first empirical studies to examine the technology acceptance of the COVID-19 Madeira Safe to Discover app by applying multidisciplinary constructs to the best of our knowledge. Still, several limitations affected the range of our results. Although we had a significant sample of several European nationalities and cultures, there was still a bias toward a specific nationality. To understand this bias’s effect, we analyzed the moderator effects of nationality in our model, which showed the same evidence of invariance measurement compared to other moderators (gender, age, etc). However, we did not record cultural and nationality differences in our sample. Previous work shows a significant impact of cultural diversity on social influence, usefulness, and behavior intention [112,113].

Another significant limitation of our study is that it involved people who traveled during the pandemic period. Given the mobility restrictions in place, the drastic reductions in travel, and the pandemic’s economic consequences, our sample could be biased. The sample accessed in this study could express different perceptions toward the COVID-19 Madeira Safe to Discover app compared to the general public. This potential bias effect limits the generalizability of this research, although the design method reduces the impact of the common method bias (CMB), which we encountered in this research, particularly for the new COVID-19 constructs. In addition, objectively measuring outcome variables separately (eg, frequency of use) will lead to results less likely to produces biases related to the measurement and methods used.

Despite the aforementioned limitations, we believe that this study advances the understanding of the intention to use mobile apps and those associated with safety concerns, such as COVID-19, and will provide a useful set of design guidelines and recommendations for the provision of mobile services with safety, security, and trust concerns to different user groups.

### Conclusion

In this research, recognizing the moderating role of demographics is especially significant. The intention to use the COVID-19 Madeira Safe to Discover app differs among demographic groups. Notably, the impact of social influence varies with gender, age, education, and social capital. We also observed a significant change in the role of the COVID-19 impact over demographics. Finally, high indicators of users’ social capital have a tremendous effect on the intention to use COVID-19 safety systems, which suggests that localized versions of these apps are likely to be more successful than general ones.

Anticipating user behavior is notoriously tricky, especially under unprecedented circumstances. An obvious direction for future work would be to apply our measurement model to a longitudinal approach on a more comprehensive technology, such as digital contact tracing. Such a study will sample a more extensive and more culturally diverse user base. This could be accomplished using quota sampling or stratified sampling to guarantee a specific demographic distribution. Longitudinal research could observe changes in the importance of constructs over time. However, a more thorough validation of the generalized application of our research model would imply a widespread data collection process. Nevertheless, this would enable examining the significant effects of safety, privacy, and trust on behavioral intention over time. Future research could also consider supplementing other precursors of behavioral intention. The results of this study could open new avenues for future research. For instance, this research model could be applied to other contexts where safety plays an important role, such as health care, and where privacy is a major concern, such as surveillance and social networking. In addition, understanding how to study the UTAUT model through more parsimonious items can reduce the overload of the questionnaires.

## Appendix

Multimedia Appendix 1 Goodness-of-fit indices.

Multimedia Appendix 2 Discriminant validity through the heterotrait-monotrait ratio.

Multimedia Appendix 3 Validity measures.

Multimedia Appendix 4 Summary of hypothesis tests.

Multimedia Appendix 5 Fit indices for invariance checks of moderator effects.

## Abbreviations

AI: artificial intelligence
AGFI: adjusted goodness-of-fit index
AVE: average variance extracted
CFA: confirmatory factor analysis
EU: European Union
HCI: human-computer interaction
HTMT: heterotrait-monotrait ratio of correlations
SEM: structural equation modelling
SMC: squared multiple correlation
SRMR: standardized root-mean-square residual
TAM: models of technology adoption
TRA: theory of reasoned action
UTAUT: unified theory of acceptance and use of technology
