# Supplementary material for: Modeling Adoption, Security, and Privacy of COVID-19 Apps: Findings and Recommendations From an Empirical Study Using the Unified Theory of Acceptance and Use of Technology
Source: JMIR Hum Factors. 2022 Sep 14;9(3):e35434. doi: 10.2196/35434 (PMC9484482; doi:10.2196/35434)
Supplement: Multimedia Appendix 1 [file humanfactors_v9i3e35434_app1.docx]

Table 1 - Fit indices for the structural model.

| **Fit statistics** | **Value** | **Recommended value** |
| --- | --- | --- |
|  |  |  |
| CHI-square / df | 5804.422 |  |
|  | df=206 |  |
|  | *P*=.001 |  |
| Goodness of Fit Index (GFI) | 0.959 | >0.9 [98] |
| Ajusted Goodness of Fit Index (GFI) | 0.928 | >0.8 [99] |
| Bentler-Bonett Normed Fit Index (NFI) | 0.958 | >0.9 [115] |
| TLI (Tucker-Lewis Index) | 0.950 | approaches 1 [115] |
| RMSEA (Root Mean Square Error Approximation) | 0.053 | <0.06 [95] |
| SMR (Standardized RMR) | 0.063 | the smaller the better the fit [109] |
