# Supplementary material for: Modeling Adoption, Security, and Privacy of COVID-19 Apps: Findings and Recommendations From an Empirical Study Using the Unified Theory of Acceptance and Use of Technology
Source: JMIR Hum Factors. 2022 Sep 14;9(3):e35434. doi: 10.2196/35434 (PMC9484482; doi:10.2196/35434)
Supplement: Multimedia Appendix 3 [file humanfactors_v9i3e35434_app3.docx]

Annex Table 3 - Validity Measures.^b^

| Measure | Impact | Safety | PerfExp | EffExp | SocInf | FacCond | IntUse | Secur | Priv | Trust |
| --- | --- | --- | --- | --- | --- | --- | --- | --- | --- | --- |
|  |  |  |  |  |  |  |  |  |  |  |
| alpha | 0.741 | 0.732 | 0.855 | 0.920 | 0.729 | 0.918 | 0.701 | 0.909 | 0.910 | 0.852 |
| CR | 0.724 | 0.728 | 0.856 | 0.920 | 0.759 | 0.918 | 0.687 | 0.907 | 0.910 | 0.852 |
| AVE | 0.470 | 0.472 | 0.749 | 0.852 | 0.588 | 0.848 | 0.523 | 0.766 | 0.835 | 0.743 |

^b^constructs abbreviated as follows - COVID-19 Impact (Impact), Safety measures (Safety), Performance Expectancy (PerfExp), Effort Expectancy (EffExp), Social Influence (SocInf), Facilitating Conditions (FacCond), Behaviour Intention to Use (IntUse), Perceived Security (Security), Privacy Risk (Privacy), Perceived Trust (Trust);

alpha: Cronbach alpha. CR: Composite reliability, AVE: Average Variance Extracted.
