# Supplementary material for: Modeling Adoption, Security, and Privacy of COVID-19 Apps: Findings and Recommendations From an Empirical Study Using the Unified Theory of Acceptance and Use of Technology
Source: JMIR Hum Factors. 2022 Sep 14;9(3):e35434. doi: 10.2196/35434 (PMC9484482; doi:10.2196/35434)
Supplement: Multimedia Appendix 4 [file humanfactors_v9i3e35434_app4.docx]

Annex Table 4 - Summary of hypothesis tests.

| Hypothesis | B | StError | Z-value | *P*-value | Beta | Supported |
| --- | --- | --- | --- | --- | --- | --- |
|  |  |  |  |  |  |  |
| **H1a.** FacCond $\to$ IntUse | 0.229 | 0.01 | 23.97 | .001 | 0.251 | Yes *P<.001* |
| **H1b.** FacCond $\to$ EffExp | 0.494 | 0.011 | 43.088 | .001 | 0.460 | Yes *P<.001* |
| **H2a.** SocInf $\to$ EffExp | 0.482 | 0.019 | 25.374 | .001 | 0.394 | Yes *P<.001* |
| **H2b.** SocInf $\to$ Security | 0.677 | 0.018 | 38.13 | .001 | 0.556 | Yes *P<.001* |
| **H2c.** SocInf $\to$ PerfExp | 0.511 | 0.021 | 24.199 | .001 | 0.438 | Yes *P<.001* |
| **H3.** PerfExp $\to$ IntUse | 0.545 | 0.013 | 42.502 | .001 | 0.611 | Yes *P<.001* |
| **H4.** EffExp $\to$ PerfExp | 0.418 | 0.013 | 32.102 | .001 | 0.438 | Yes *P<.001* |
| **H5a.** Security $\to$ Trust | 0.667 | 0.012 | 54.268 | .001 | 0.703 | Yes *P<.001* |
| **H5b.** Security $\to$ IntUse | 0.147 | 0.009 | 15.922 | .001 | 0.172 | Yes *P<.001* |
| **H6a.** Privacy $\to$ Security | -0.254 | 0.009 | -29.801 | .001 | -0.301 | Yes *P<.001* |
| **H6b.** Privacy $\to$ Trust | 0.012 | 0.008 | 1.528 | **.127** | 0.015 | **No** |
| **H6c.** Privacy $\to$ PerfExp | -0.078 | 0.007 | -11.588 | .001 | -0.096 | Yes *P<.001* |
| **H7a.** Trust $\to$ PerfExp | 0.080 | 0.01 | 8.376 | .001 | 0.079 | Yes *P<.001* |
| **H7b.** Trust $\to$ EffExpe | 0.109 | 0.01 | 10.408 | .001 | 0.103 | Yes *P<.001* |
| **H8a.** Impact $\to$ Safety | 0.461 | 0.01 | 44.212 | .001 | 0.668 | Yes *P<.001* |
| **H8b.** Impact $\to$ Trust | 0.045 | 0.009 | 4.781 | .001 | 0.047 | Yes *P<.001* |
| **H9a.** Safety $\to$ Security | 0.212 | 0.015 | 14.258 | .001 | 0.148 | Yes *P<.001* |
| **H9b.** Safety $\to$ IntUse | 0.196 | 0.011 | 17.152 | .001 | 0.159 | Yes *P<.001* |
