# Supplementary material for: Modeling Adoption, Security, and Privacy of COVID-19 Apps: Findings and Recommendations From an Empirical Study Using the Unified Theory of Acceptance and Use of Technology
Source: JMIR Hum Factors. 2022 Sep 14;9(3):e35434. doi: 10.2196/35434 (PMC9484482; doi:10.2196/35434)
Supplement: Multimedia Appendix 5 [file humanfactors_v9i3e35434_app5.docx]

Annex Table 5 - Fit indices for the invariance checks of moderator effects, including configural, scalar and residual.

|  |  | $X{}^{2}$ (df) | CFI | TLI | RMSEA | SRMR |
| --- | --- | --- | --- | --- | --- | --- |
|  |  |  |  |  |  |  |
| **Age** | Configural | 3181.436 | 0.983 | 0.979 | 0.037 | 0.042 |
|  | Measurement | 3248.411 | 0.983 | 0.980 | 0.037 | 0.042 |
|  | Residual | 3333.369 | 0.983 | 0.980 | 0.037 | 0.043 |
| **Gender** |  |  |  |  |  |  |
|  | Configural | 6540.160 | 0.957 | 0.947 | 0.055 | 0.061 |
|  | Measurement | 6642.040 | 0.956 | 0.948 | 0.054 | 0.062 |
|  | Residual | 6815.243 | 0.955 | 0.949 | 0.053 | 0.062 |
| **Nationality** |  |  |  |  |  |  |
|  | Configural | 3488.449 | 0.987 | 0.984 | 0.032 | 0.044 |
|  | Measurement | 3847.368 | 0.986 | 0.983 | 0.033 | 0.046 |
|  | Residual | 4530.430 | 0.982 | 0.980 | 0.036 | 0.048 |
| **Regularity** |  |  |  |  |  |  |
|  | Configural | 3286.485 | 0.983 | 0.980 | 0.037 | 0.043 |
|  | Measurement | 3471.542 | 0.982 | 0.979 | 0.037 | 0.044 |
|  | Residual | 3962.323 | 0.980 | 0.977 | 0.039 | 0.046 |
| **Education** |  |  |  |  |  |  |
|  | Configural | 3130.900 | 0.983 | 0.980 | 0.037 | 0.042 |
|  | Measurement | 3193.448 | 0.983 | 0.980 | 0.037 | 0.042 |
|  | Residual | 3229.304 | 0.983 | 0.980 | 0.036 | 0.042 |
